# Total cholesterol concentration predicts the effect of plasmapheresis on hypertriglyceridemic acute pancreatitis: a retrospective case–control study

**DOI:** 10.1186/s12876-020-01572-w

**Published:** 2021-01-06

**Authors:** Zhu Chen, Xiaolong Huang, Na Han, Yanxia Guo, Jing Chen, Yaogui Ning, Minwei Zhang

**Affiliations:** 1 Intensive Care Unit, The First Affiliated Hospital of Xiamen University, NO. 55, Zhenhai Road, Siming District, Xiamen City, Fujian 361003 People’s Republic of China; 2 Xiamen Blood Center, NO. 121, Hubin South Road, Siming District, Xiamen City, Fujian 361004 People’s Republic of China

**Keywords:** Total cholesterol, Plasmapheresis, Effectiveness, Hypertriglyceridemia, Acute pancreatitis

## Abstract

**Background:**

What kind of patients with hypertriglyceridemic acute pancreatitis (HLAP) might benefit from plasmapheresis (PP) remains unknown. The objective of this study is to determine the predict function of total cholesterol (TC) on the Triglyceride (TG)-lowing effect in patients on either non-PP or PP therapy.

**Methods:**

Patients were categorized into high total cholesterol (HTC)/low total cholesterol (LTC) groups based on TC level of 12.4 mmol/L. The primary outcome was TG reduction to below 500 mg/dL within 48 h. Linear mixed-effect model and logistic regression analyses were used to assess the association of TC level and TG-lowing efficacy in different therapy groups.

**Results:**

Compared with LTC group, patients with HTC showed more severe imaging manifestations (*p* < 0.001) and higher APACH II scores (*p* = 0.036). Deaths occurred only in HTC groups. Significant interaction of time sequence with the 2 TGs-lowing therapy groups on TG level was only found in HTC group (*p* < 0.001). In patients with elevated TC level, primary outcome occurred in 66.67% of patients in the PP group, and 27.91% in the non-PP group. After adjustment for age, gender, CT grade and APACH II score, the odd ratio remain significant (OR 5.47, 95% confidence interval [CI] 1.84–16.25, *p* = 0.002). Furthermore, in patients with lower TC level, no significant difference was found in primary outcome between PP group and non-PP group (81.25% versus 62.30%, adjusted OR 2.05; 95% CI 0.45–9.40; *p* = 0.353).

**Conclusions:**

TC could be a potential biomarker to predict the effects of TG-lowing therapy in patients with HLAP.

## Background

Acute pancreatitis (AP) is a clinically common life-threatening disease, and its incidence is increasing worldwide [1]. Hypertriglyceridemia is the third most common cause of AP after the gallstones (up to 60%) and alcohol (30%), accounting for nearly 10% of all cases. More importantly, there may be a dose–response relationship between hypertriglyceridemia and the severity and complications of AP [2]. The incidence of hypertriglyceridemic acute pancreatitis (HLAP) is gradually increasing [3, 4], and its associated mortality can exceed 30% [5].

Basic treatments for HLAP are strict restrictions regarding oral fasting, fluid resuscitation and analgesia. Beyond that, plasmapheresis (PP) has also been recommended for the treatment of HLAP due to its rapid removal of abnormal substances from patients' blood [6, 7].

At present, the clinical efficacy of PP for patients with HLAP is still unclear and controversial. Some studies report that PP can remove triglycerides (TGs) and chylomicron from circulation drastically within hours [8], while a recent retrospective study found that PP therapy had no additional TG-lowering effects [9] What’s more, many studies have found that PP does not reduce mortality rates among patients with HLAP [10, 11]. In addition, PP requires special treatment equipment, is expensive, and involves some risks, such as allergy, bleeding and infection [12].

The vast heterogeneity of the underlying clinical scenarios of patients with HLAP partly leads to TG-lowering treatment failure. Therefore, identifying the patients who are more suitable for PP treatment is important, but few relevant studies have been published. Total cholesterol (TC) can act as an early predictor of persistent organ failure and mortality in patients with AP [13, 14]. Additionally, TC are favorable predictors of the development of severe acute pancreatitis (SAP) in patients with AP [15]. Accordingly, we propose that TC levels might be useful to predict patients’ response to PP therapy.

## Methods

This retrospective case–control study was approved by the institutional review board of the first affiliated hospital of Xiamen University.

### Patients

Between January 2013 and December 2018, 1772 patients were diagnosed with pancreatitis and received treatment at the first affiliated hospital of Xiamen University. (Xiamen, China). In order to avoid selection bias as much as possible, we continuously included patients of 5 years. A total of 150 patients were diagnosed with HLAP and included in this retrospective case–control study. 46 patients were treated with PP and 104 patients were not. HLAP was diagnosed using the Atlanta Classification criteria [3]. The inclusion criteria were as follows: (1) TGs > 1000 mg/dL; (2) the occurrence of two in the following three factors: typical abdominal pain, pancreatic enzymes exceeding the normal upper limit by more than 3 times, and radiological findings of AP [16]; (3) age of 18 years or older. We excluded pregnant women with HLAP and patients with other causative conditions, such as alcohol, gall stones, trauma or neoplasm.

### Treatment

The initial treatment of HLAP, as with other causes of pancreatitis, includes intestinal rest, fasting, intravenous fluids and pain relief. PP will be performed as soon as possible within 24 h after admission to ICU because of severe AP when the patient's TG level was higher than 1000 mg/dL and informed consent. The PP sessions were performed using the Aquarius system (Edwards Lifesciences LLC, One Edwards Way, Irvine, USA) and the Plasmaflo TPE op-08w filter (Asahi KASEI Medical Co., Ltd, Yurakucho, Chiyoda-ku, Tokyo). In general, approximately 3 L of plasma were exchanged at a time, and the treatments lasted approximately 3 h. During PP, heparin (500 U/h) was continuously pumped before the filter, and calcium (1 g/h) was added after the filter. PP was performed once a day until TG levels were below 1000 mg/dL.

### Outcome

The primary efficacy outcome was TGs < 500 mg/dL within 48 h of admission [17]. The secondary efficacy outcome included the change in serum TG concentrations within 72 h of admission.

### Data collection

Demographic, clinical, and laboratory data were collected on the day of admission. Demographic data included age, sex and body mass index (BMI). Clinical variables included hypertension, diabetes mellitus and diabetic ketosis, hyperuricemia and medical history of pancreatitis. Baseline levels of TC, HDL-C, LDL-C, prealbumin, albumin, serum calcium, C-reactive protein, amylase and lipase were also collected within 24 h of admission. The time course of serum TG concentration (at baseline, 24 h, 48 h and 72 h) was involved in the results analysis. The severity of HLAP was assessed by the Ranson score, the APACHE II score, the Balthazar CT grade and organ function [18]. We simultaneously collected data regarding medical treatment for hypertriglyceridemia (insulin/heparin), complications or comorbidities of HLAP, length of hospitalization, hospital charges, and mortality at 28 days and 90 days.

### Statistical analysis

Statistical analyses were performed using R for Windows (version 3.4.2, http://www.r-project.org/). The data are presented as the median (interquartile range) or number (%). Patients were categorized into high total cholesterol (HTC) /low total cholesterol (LTC) groups based on a TC level of 12.4 mmol/L (2 times the ‘high’ limit of total cholesterol based on the recommendations of the National Heart, Lung, and Blood Institute (NHLBI) of the United States [19]). Categorical variables were compared by chi-square/Fisher’s exact tests. Because of skewed distribution, continuous variables were compared by the Mann–Whitney U test. For LTC and HTC group, the difference between PP and non-PP therapy in the rates of TG < 500 mg/dL within 48 h of admission was analyzed by using univariate and multivariate logistic regression analyses. We included predictors with a *p* value < 0.1 from univariate analysis into the backward stepwise multivariate regression with the Akaike Information Criterion (AIC). Treatment group (PP group and non-PP group)-time sequence interactions were assessed using the linear mixed-effect model according to repeated measures of TGs. After classifying the patients into four groups based on TC and TG-lowing therapy, we used Fisher’s exact tests and Kruskal–Wallis test to compare categorical and continuous variables. *p* value were adjusted by Bonferroni correction for multiple comparisons among the defined groups. A 2-sided *p* value of less than 0.05 was considered to indicate statistical significance.

## Results

### Patient characteristics

A total of 150 patients were included in this study. 46 patients received therapeutic PP, and 104 patients did not. The baseline clinical characteristics are listed in Table 1. The median age was 38 years. Most of the patients were male (78.67%) and had a high BMI (median 26.15; IQR 24.20–28.30). 39.33% of the patients had history of pancreatitis. The median plasma levels of TGs and TC were 1760.2 mg/dL and 12.28 mmol/L, respectively. More than half (51.33%) of the patients showed more fluid leakage on imaging (Balthazar CT grade: D/E). Organ dysfunction occurred in 20% of all patients. 67.3% and 56.0% patients were treated with insulin and heparin for reducing plasma TGs, respectively. A total of four deaths were associated with AP, including two cases of sudden intraperitoneal hemorrhage, one case of systemic inflammation associated with pancreatitis resulted in multiple organ dysfunction, and one case of peripancreatic infection with septic shock.

**Table 1:** Baseline clinical characteristics

	All (n = 150)	LTC group (cholesterol ≤ 12.40 mmol/L, n = 77)	HTC group (cholesterol > 12.40 mmol/L, n = 73)	*p*
Baseline characteristics				
Age (years)	38.00 (31.00–44.00)	36.00 (31.00–43.00)	40.00 (33.00–45.00)	0.047
Male	118 (78.67%)	66 (85.71%)	52 (71.23%)	0.049
BMI (kg/m^2^)	26.15 (24.20–28.30)	26.00 (24.80–28.40)	26.40 (24.20–28.30)	0.880
Hypertension	23 (15.33%)	10 (12.99%)	13 (17.80%)	0.553
Diabetes	44 (29.33%)	20 (25.97%)	24 (32.88%)	0.454
History of pancreatitis	59 (39.33%)	33 (45.21%)	25 (34.25%)	0.282
TG (mg/dL)	1760.20 (1187.60–2534.30)	1599.10 (1140.70–1981.40)	2224.80 (1351.30–3291.20)	< 0.001
TC (mmol/L)	12.28 (8.83–15.96)			
HDL-C (mmol/L)	0.97 (0.59–2.86)	0.97 (0.61–2.37)	0.93 (0.58–4.23)	0.347
LDL-C (mmol/L)	2.86 (1.55–5.30)	1.91 (1.18–3.31)	4.50 (2.13–7.23)	< 0.001
Ca (mmol/L)	2.17 (1.98–2.48)	2.24 (2.05–2.48)	2.11 (1.95–2.59)	0.183
Amylase (U/L)	202.00 (108.20–391.80)	246.00 (110.00–373.00)	182.00 (84.00–396.00)	0.330
Lipase (U/L)	627.80 (290.30–1513.20)	588.00 (288.10–1496.00)	637.00 (297.60–1592.00)	0.867
Severity of hypertriglyceridemic pancreatitis				
Balthazar CT grade				< 0.001
< D	73 (48.67%)	48 (62.33%)	25 (34.25%)	
≥ D	77 (51.33%)	29 (37.66%)	48 (65.75%)	
Organ function				0.111
NO	120 (80.00%)	66 (85.71%)	54 (73.97%)	
YES	30 (20.00%)	11 (14.29%)	19 (26.03%)	
Ranson score				0.396
< 3	130 (86.67%)	69 (89.61%)	61 (83.56%)	
≥ 3	20 (13.33%)	8 (10.39%)	12 (16.44%)	
APACHE II	7.00 (5.00–9.00)	7.00 (5.00–9.00)	8.00 (6.00–10.00)	0.036
Treatment				
Insulin	101 (67.33%)	51 (66.23%)	50 (68.49%)	0.904
Heparin	84 (56.00%)	43 (55.84%)	41 (56.16%)	1
AP-associated death	4 (2.67%)	0 (0%)	4 (5.48%)	0.054

BMI, body mass index; TG, triglyceride; TC, total cholesterol; HDL-C, high-density lipoprotein cholesterol; LDL-C, low-density lipoprotein cholesterol; Ca, calcium. AP, acute pancreatitis

### Baseline characteristics of total cholesterol subgroup of patients

As shown in Table 1, patients were categorized into 2 groups based on a TC level of 12.4 mmol/L. Patients in the HTC group were older (*p* = 0.047) and has lower proportion of male (*p* = 0.049). In terms of disease severity assessment, patients in the HTC group had more patients with Balthazar CT grade ≥ D (*p* < 0.001) and higher APACHE II score (*p* = 0.036). However, there was no significant difference in BMI, proportion of diabetes, HDL-C concentration, calcium level, blood amylase/lipase level, insulin/heparin therapy and organ dysfunction between the two groups. The AP-associated death in HTC group was higher than that in LTC group (5.48% vs. 0%), but there was no statistical difference (*p* = 0.054).

### Effects of plasmapheresis on primary outcomes by TC categories

**Fig. 1:**
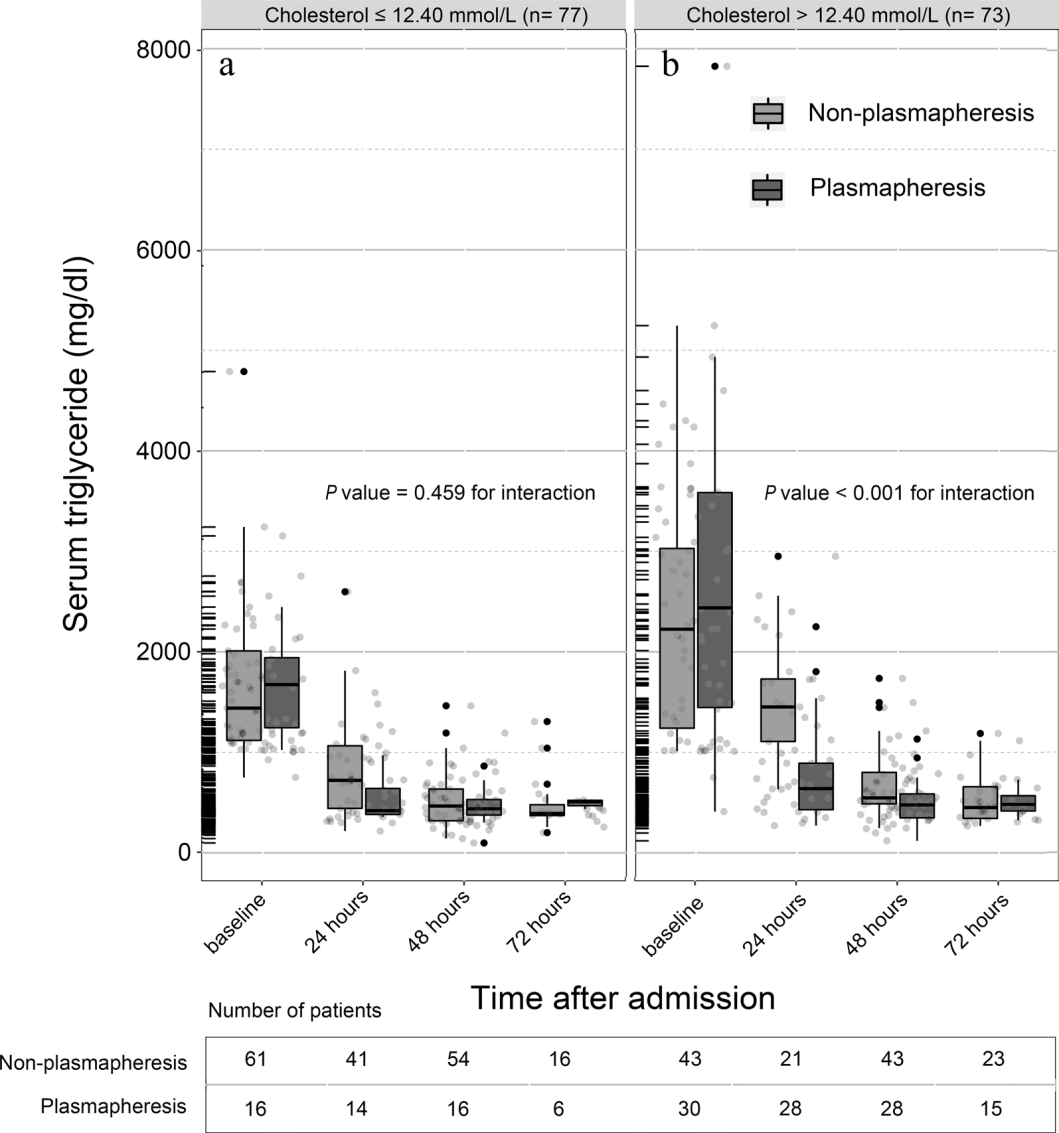
Time course of the serum triglyceride concentration changes within 72 h after admission by total cholesterol levels. **a** Time course of triglyceride changes in the low total cholesterol group. Compared with the effects observed in the non-plasmapheresis group, plasmapheresis did not reduce triglyceride more effectively (*p* = 0.459). **b** Time course of triglyceride changes in the high total cholesterol group. Compared with the effects observed in the non-plasmapheresis group, plasmapheresis reduced the triglyceride concentration more effectively (*p* < 0.001)

There was significant difference of PP’ curative effect between HTC and LTC groups in reducing TG level to below 500 mg/dL within 48 h (Table 2). In patients with TC levels higher than 12.4 mmol/L, PP therapy decreased TGs more effectively (OR: 5.17; 95% CI: 1.88–14.19, *p* = 0.001); however, in patients with lower TC levels, there was no significant difference in the rate of the primary outcome (*p* = 0.164). After adjusting for age, sex, Balthazar CT grade and APACHE II score, the outcome remained consistent (*p* = 0.002 for HTC group and *p* = 0.353 for LTC group). Furthermore, the time course of the lowering of TGs within 72 h of admission categorized by TC-level groups is depicted in Fig. 1. There was no significant interaction effect between time and the treatment group (*p* = 0.459 for interaction) in the LTC group. However, we did find a significant association between rapidly reduction of TGs and PP therapy (*p* < 0.001 for interaction) in the HTC group.

**Table 2:** Effects of plasmapheresis on primary outcomes by TC categories

	Plasmapheresis, event rate (%)^a^	Non-plasmapheresis, event rate (%)	Crude		Multivariable adjusted^b^	
			OR (95%CI)	*p* value	OR (95%CI)	*p*
TC (mmol/L)						
≤ 12.40 (n = 77)	13 (81.25%)	38 (62.30%)	2.62 (0.67–10.20)	0.164	2.05 (0.45–9.40)	0.353
> 12.40 (n = 73)	20 (66.67%)	12 (27.91%)	5.17 (1.88–14.19)	0.001	5.47 (1.84–16.25)	0.002

TC, total cholesterol

^a^TG ≤ 500 mg/dl within 48 h

^b^Adjusted for age, sex, Balthazar CT grade and APACHE II score

### Clinical outcomes of patients by TC categories and treatment group

The influence of PP to patients’ clinical outcome may be modified by TC levels. Accordingly, patients were divided into four groups based on their TC levels and whether they had been treated with PP or not. As shown in Table 3, 61 patients were classified into group 1 (LTC + non-PP), 16 into group 2 (LTC + PP), 43 into group 3 (HTC + non-PP), and 30 into group 4 (HTC + PP). Compared the group 1 and group 3, patients in group 2 and group 4 had higher effective rate in reducing TGs. And non-PP therapy seems less effective in reducing TGs in patients with TC level higher than 12.40 mmol/L. Patients with PP treatment had longer hospital stays and higher hospital costs. Deaths only reported in groups 3 and group 4, with 90-day mortality rates of 2.33% and 10.00%, respectively.

**Table 3:** Clinical outcomes of patients by TC categories and treatment group

	Group 1 TC ≤ 12.40 mmol/L + non PP (n = 61)	Group 2 TC ≤ 12.40 mmol/L + PP (n = 16)	Group 3 TC > 12.40 mmol/L + non PP (n = 43)	Group 4 TC > 12.40 mmol/L + PP (n = 30)	*p*
TG ≤ 500 mg/dl within 48 h	38 (62.30%)^#^	13 (81.25%)^‡^	12 (27.91%)^#,^^‡,ξ^	20 (66.67%)^ξ^	< 0.001
Hospital stay (days)	8.00 (6.00–9.00)^*,^^†^	16.00 (10.00–21.50)^*,‡,¶^	10.00 (7.00–12.50)^‡^	11.00 (8.25–16.75)^†,¶^	< 0.001
Hospital costs (Yuan in thousand)	10.92 (8.69–14.37)^*,^^†^	52.55 (30.89–91.59)^*,‡^	14.75 (12.01–21.34)^‡,^^ξ^	40.17 (35.30–55.88)^†,ξ^	< 0.001
28-day mortality	0 (0%)	0 (0%)	1 (2.33%)	1 (3.33%)	0.438
90-day mortality	0 (0%)^†^	0 (0%)	1 (2.33%)	3 (10.00%)^†^	0.039

^*^p < 0.05 for groups 1 versus 2

^#^*p* < 0.05 for groups 1 versus 3

^†^*p* < 0.05 for groups 1 versus 4

^‡^*p* < 0.05 for groups 2 versus 3

^¶^*p* < 0.05 for groups 2 versus 4

^ξ^*p* < 0.05 for groups 3 versus 4

**Fig. 2:**
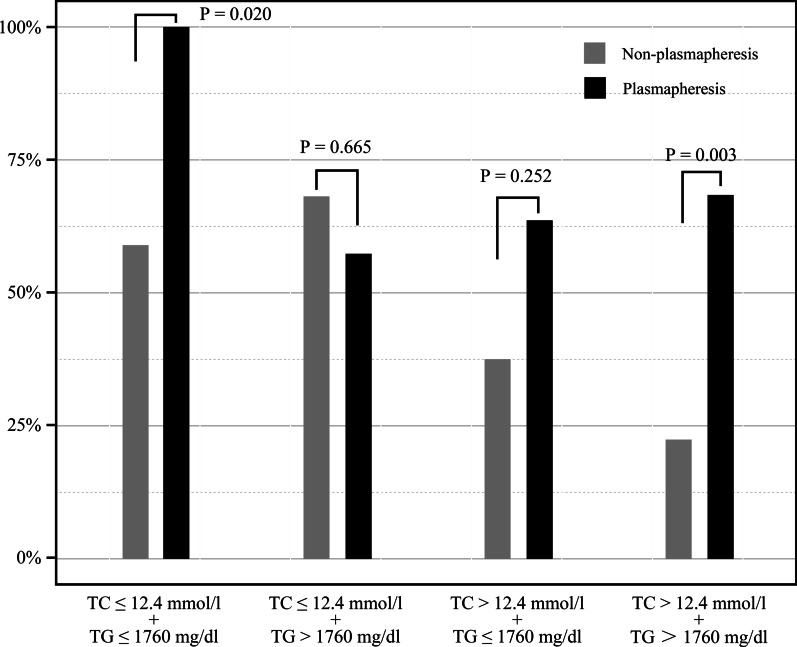
Effect of plasmapheresis and non-plasmapheresis in different triglyceride and total cholesterol combination groups. TC, total cholesterol; TG, triglyceride

To evaluate whether the TG-lowing effect is affected by baseline TG, patients were categorized into high triglyceride (HTG) /low triglyceride (LTG) groups based on a TG level of 1760 mg/dl (median value of TG). On the basis of TG grouping, the patients were further divided into LTC + LTG/LTC + HTG/HTC + LTG/HTC + HTG groups according to the level of TC (cutoff:12.4mmo/L). To explore the curative effect of PP, we itemized each group into subgroups of PP and non-PP. As depicted in Fig. 2, PP was shown to be more effective in reducing TGs below 500 mg/dL in the LTC + LTG and HTC + HTG groups within 48 h. PP also has the potential in lowering TGs in the HTC + LTG group, although the *p* value exceeded 0.05. Intriguingly, its seems that patients in LTC/HTG group showed a better reduction of TG by non-PP therapy.

## Discussion

In patients with HLAP, different TC level can result in different therapeutic effects of TG-lowing treatments in clinical application. To our knowledge, this is the first study to explore the association between TC levels and PP therapy among patients with HLAP.

Previous studies have demonstrated that a higher level of TGs might contribute to more severe pancreatitis [17, 20, 21]. Reducing TG levels as soon as possible is an important strategy for the treatment of HLAP. PP has traditionally been considered an alternative to TG clearance. In addition, the American Society for Apheresis (ASFA) recommends PP as an indication for HLAP (grade 2C evidence) [22].

Many studies have shown that high TG levels might be associated with disease progression, and PP should be performed as early as possible when the patient is diagnosed with HLAP [8, 23]. However, a few previous studies with limited sample sizes have shown that PP was not more effective in reducing TG concentrations [9]. And conservative treatment is effective and safe without PP [24]. Such controversies may be related to the surrogate endpoint, instead of clinical outcomes to evaluate the effect of TG-lowing therapy, in which variability and inconsistence with clinical outcome have been shown.

Until now, what kind of HLAP patients are much more appropriate for PP treatment remains unclear. Early identification of patients suitable for PP is of significant clinical importance [25]. Our study has a relatively large sample size with long time span, which enhances the reliability of the results. In our study, we found that the plasma TC concentration in most patients far exceeded the ‘high’ limit recommended by the NHLBI. After further grouping by TC concentration, PP showed more efficacy in reducing TGs in the HTC group but not in LTC group. Further considering the impact of baseline TG, patients with HTG and HTC were most suitable for PP treatment, while patients with HTG and LTC were least suitable. Lipid metabolism is dramatically complex and varies from person to person. Common risk factors, such as age, gender, insulin/heparin treatment, Balthazar CT grade and severity of illness, are known to interfere with TG-lowing treatment [26]. We have adjusted these factors and found that TC remained have an independent effect on TG-lowing therapy and the outcomes.

In addition, primary genetic variations among patients are also an important factor of affecting lipid metabolism [27, 28]. However, it is time-consuming and expensive to perform genetic screening. In contrast, measuring blood biomarkers such as TC level was practical, easy to operate, and economical to predict a patient’s response to TG-lowing therapy.

Bile acids are synthesized from TC in the liver and play an important role in the solubilization of lipids in the intestine by acting as biological detergents. Bile acids also affect the absorption of dietary fat, and perturbing bile acid production results in reduced lipid absorption [29]. On the other hand, many enzymes or receptors, such as glycerol-3-phosphate acyltransferase 3, acyl-CoA synthase 5, liver X receptors and farnesoid X receptor, are involved in the metabolism of TC and TGs, and there is a mutual feedback regulatory effect [30]. The mutual feedback regulatory mechanism between TC and TGs has not been fully understood, and further research is needed.

Previous studies have shown that the release of systemic inflammatory factors is involved in the occurrence and development of AP [31, 32]. HTC not only leads to a severe inflammatory state, it was an independent risk factor for SAP development [33, 34]. Evidence has unveiled that HTC can lead macrophages and other immune cells to release a large amount of inflammatory factors by enhancing Toll-like receptor (TLR) signaling [35]. The amplification of the inflammatory process can lead to pancreatitis-associated organ injury [36, 37]. Furthermore, through the nuclear transcription factor kappa-B (NF-kB) signaling pathway, HTC can also increase the release of oxygen free radicals, induce lipid peroxidation, damage endothelial cells, and cause further organ dysfunction [32, 38, 39]. Moreover, many animal experiments have shown that HTC can also produce more inflammatory cells by promoting the maturation of the bone marrow and spleen [40]. PP can rapidly remove a variety of inflammatory factors and antibodies, reduce systemic inflammatory reactions and alleviate organ dysfunction [41, 42]. Patients with elevated TC level may benefit from PP therapy. However, it cannot be fooled that PP has the disadvantages of higher costs, longer hospital stays, and more complicated operations.

Our study has several limitations. Firstly, it was a retrospective observational study, the PP group included more severe patients. However, after adjustment for conventional confounding factors, the results remained consistent. Secondly, no gene analysis was performed to determine whether the clinical effects of PP were related to the patient's genotype. Thirdly, cytokine levels were not detected, so the relationship between the efficiency of PP in removing inflammatory cytokines and prognosis was unclear. Until now, whether patients with HLAP can benefit from PP remains unclear, and further randomised controlled trials (RCTs) are needed to evaluate the mortality in the HLAP treated with and without PP. There is an ONGOING RCT study that hopes to help answer this question [43].

## Conclusions

Baseline TC level may predict the effect of conservative treatment or PP therapy on TG-lowing in HLAP patients. TC may also serve as an adjunctive biomarker for clinical selection of patients more suitable for PP.

## Data Availability

The datasets used and/or analysed during the current study are available from the corresponding author on reasonable request.

## Abbreviations

PP: Plasmapheresis
HLAP: Hypertriglyceridemic acute pancreatitis
TC: Total cholesterol
AP: Acute pancreatitis
TG: Triglyceride
HTG: Hypertriglyceridemia
BMI: Body mass index
HDL-C: High-density lipoprotein cholesterol
LDL-C: Low-density lipoprotein cholesterol
Ca: Calcium
OR: Odd ratios
CI: Confidence interval
